# A *Drosophila* Model Reveals the Potential Role for *mtt* in Retinal Disease

**DOI:** 10.3390/ijms25020899

**Published:** 2024-01-11

**Authors:** Wenfeng Chen, Wenmiao Zhong, Lingqi Yu, Xiang Lin, Jiayu Xie, Zhenxing Liu

**Affiliations:** 1 Institute of Life Sciences, College of Biological Science and Engineering, Fuzhou University, Fuzhou 350108, China; 2 School of Medicine, Chongqing University, Chongqing 400044, China; gtxiejy@stu.cqu.edu.cn; 3 State Key Laboratory of Resource Insects, Institute of Apicultural Research, Chinese Academy of Agricultural Sciences, Beijing 100093, China

**Keywords:** *Drosophila*, *mtt*, GRM6, CSNB, retina, eye

## Abstract

Congenital stationary night blindness (CSNB) is a genetically heterogeneous inherited retinal disorder, caused by over 300 mutations in 17 different genes. While there are numerous fly models available for simulating ocular diseases, most are focused on mimicking retinitis pigmentosa (RP), with animal models specifically addressing CSNB limited to mammals. Here, we present a CSNB fly model associated with the *mtt* gene, utilizing RNA interference (RNAi) to silence the *mtt* gene in fly eyes (homologous to the mammalian GRM6 gene) and construct a CSNB model. Through this approach, we observed significant defects in the eye structure and function upon reducing *mtt* expression in fly eyes. This manifested as disruptions in the compound eye lens structure and reduced sensitivity to light responses. These results suggest a critical role for *mtt* in the function of fly adult eyes. Interestingly, we found that the *mtt* gene is not expressed in the photoreceptor neurons of adult flies but is localized to the inner lamina neurons. In summary, these results underscore the crucial involvement of *mtt* in fly retinal function, providing a framework for understanding the pathogenic mechanisms of CSNB and facilitating research into potential therapeutic interventions.

## 1. Introduction

Congenital stationary night blindness (CSNB) refers to a non-progressive retinal disorder determined by genetic factors, exhibiting high clinical and genetic heterogeneity [1]. It is primarily caused by mutations in protein-coding genes related to signal processing within photoreceptors or signal transmission through retinal bipolar cells [2]. Patients with this condition typically experience difficulty in visual acuity under low light conditions, delayed adaptation to changes between light and dark, and light-sensitive reactions. Ocular signs often include high myopia, strabismus, and nystagmus [3]. Based on electroretinogram responses, CSNB can be classified into two types: Riggs CSNB, associated with defects in rod photoreceptors, and Schubert–Bornschein CSNB, related to signal transmission from photoreceptors to bipolar cells [3,4]. Schubert–Bornschein CSNB can be further categorized into complete congenital stationary night blindness and incomplete congenital stationary night blindness. To date, there are over 300 mutated genes associated with CSNB [5], including Leucine-rich Repeat, Immunoglobulin-like and Transmembrane Domains Protein 3 (LRIT3), nyctalopin (NXY), transient receptor potential melastatin 1 (TRPM1), G protein receptor 179 (GPR179), and metabotropic glutamate receptor 6 (mGluR6/GRM6). For instance, mutations in these genes, such as LRIT3, NXY, TRPM1, GPR179, and GRM6, have been identified, each playing a significant role in the pathogenesis of CSNB.

In the process of signal transmission in the retina, bipolar cells play the role of conveying light-driven signals to the retinal output ganglion cells [6]. The mGluR6 protein is located at the dendritic tips of retinal bipolar cells. In the absence of light, glutamate released in the synaptic cleft activates mGluR6, initiating the ON-Bipolar cell (ON-BC) signal cascade, leading to the closure of the ion channel transient receptor potential melastatin 1 (TRPM1). Conversely, in response to light, fewer glutamate molecules bind to mGluR6, resulting in the opening of the TRPM1 channel [7]. GRM6^nob^ mice represent a series of mouse models with GRM6 mutations causing CSNB. Currently, five CSNB disease models have been described in mice: Grm6tm1Nak (referred to as Grm6^−/−^), nob3, nob4, nob7, and nob8. Similar to CSNB patients [2,8,9,10,11], the majority of Grm6 mutant mice exhibit a lack of b-waves under dark-adapted conditions in electroretinography (ERG). Additionally, these mice show a deficiency in the mGluR6 protein.

Over the past two decades, research on CSNB mutant mice has significantly advanced our understanding of retinal signal transduction mechanisms and the interactions of ON-BC proteins. *Drosophila*, the fruit fly, is a genetically tractable model organism. Studies have shown that 75% of known pathogenic genes in humans have counterparts in flies [12], making the genetically straightforward fly an excellent candidate for research on human genetics and disease mechanisms. The fly has been widely used to model diseases related to human brain neurodevelopment [13]. Additionally, its short lifespan, high reproductive rate, mature genetic manipulation techniques, and diverse mutant varieties provide convenience for research. Detailed analyses of fly mutants and studies on misexpression have identified numerous genes encoding transcription factors (such as Pax6/eyeless) and components of signaling pathways (such as Notch and Hedgehog signaling) that are crucial for eye development [12,13]. Interestingly, nearly all of these genes have orthologs in vertebrates, many of which are also associated with eye development, collectively regulating fly eye development [14]. In the light transduction pathway of flies, the signaling cascade in photoreceptor cells and intrinsic photosensitive retinal ganglion cells (ipRGCs) exhibits similarities [15]. Light stimulation causes melanin in cells to absorb light, activating the inositol phosphate cascade reaction, leading to the opening of DAG-sensitive TRPC 6/7 channels [16,17]. Furthermore, TRPM1 is an essential cation-gated channel protein in vertebrate bipolar cells, and its association with CSNB is established [18]. Therefore, studying the TRPC 6/7 channels in flies contributes to understanding the mechanism of the ON-BC pathway in vertebrates [19]. In 1910, Morgan discovered the first eye mutation in flies when normal red-eyed flies turned white-eyed. Subsequently, the first study proving that a mutation in the rhodopsin gene (Rh1) led to retinal degeneration was reported in the 1980s [20]. To date, six instances of these gene mutations in flies causing retinal degeneration have been documented.

In this study, we establish a fly model of CSNB by specifically reducing the expression of the homologous gene *mtt* and *mGluR*, corresponding to the mammalian GRM6. Early research reports that mtt (mangetout, also known as DmX) is an orphan G protein-coupled receptor [21]. The disruption of the *mtt* gene inhibits the fly’s avoidance response to toxins [22]. However, whether the *mtt* gene plays a role in eye function has not been previously reported. Here, we demonstrate that RNA interference (RNAi)-induced reduction of *mtt* but not *mGluR* expression in the eyes significantly disrupts the normal compound eye structure and function in flies. This phenotype is similar to those observed in CSNB patients with mutations in the homologous gene GRM6. Invertebrate models of CSNB may contribute to our understanding of the signaling mechanisms in bipolar cells and facilitate high-throughput screening of potential therapeutic drugs for such disorders.

## 2. Results

### 2.1. The Fly Genes mtt and mGluR Are Highly Homologous to the Human Gene GRM6

**Figure 1:**
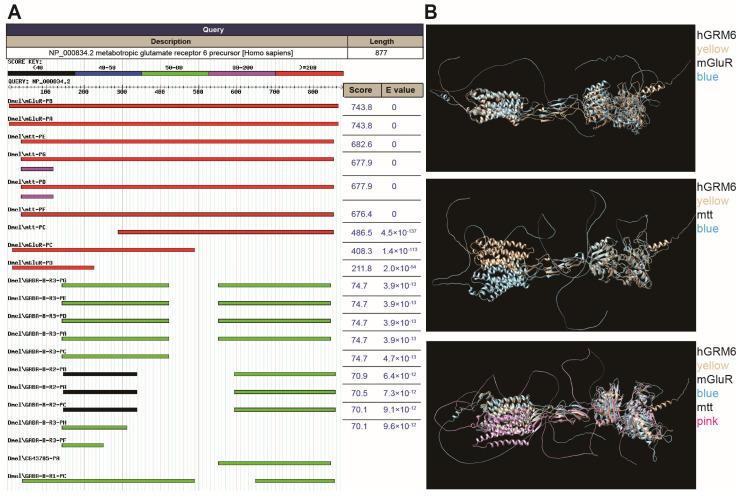
The fly genes *mtt* and *mGluR* exhibit high similarity to the human gene GRM6. (**A**) Sequence alignment of human GRM6 and *Drosophila* mtt/mGluR proteins. (**B**) Pairwise comparison of different proteins via a 3D structure diagram of human GRM6 (hGRM6) and *Drosophila* (mtt/mGluR).

In order to construct a fly model simulating CSNB caused by GRM6 deficiency, we identified homologous genes in flies—*mtt* and *mGluR*—aligned with the human GRM6 through the Flybase database (http://flybase.org/, accessed on 2 October 2023) (Figure 1A). We initially assessed their conservation, and notably, we found that fly mtt and mGluR and their human counterparts are highly conservative. The overall sequence similarity between the protein sequence of human GRM6 and the protein sequences of fly mtt and mGluR is approximately 60% (Figure S1). Furthermore, their three-dimensional protein structures also exhibit a considerable degree of similarity (Figure 1B).

### 2.2. Strain Selection for RNAi-Mediated Reduction of mtt and mGluR Expression in the Fly Head

**Figure 2:**
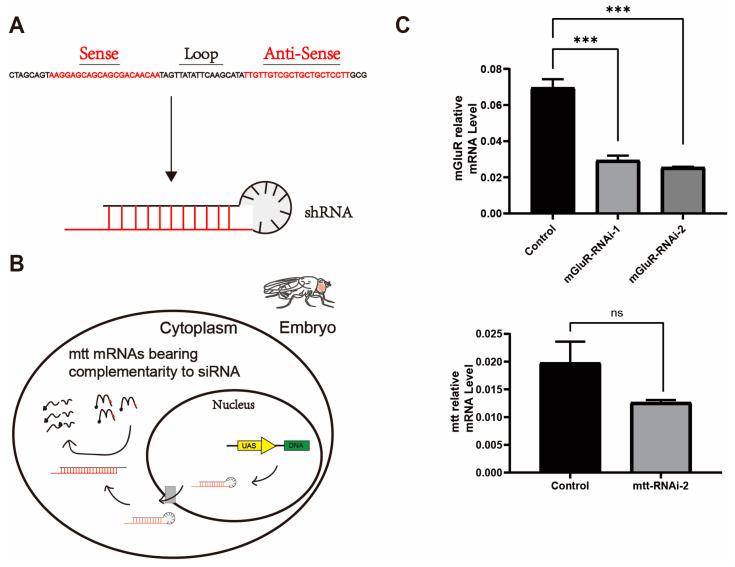
Reduction of *mGluR*/*mtt* expression through RNAi-mediated silencing. (**A**) The structure diagram of shRNA was constructed with *mtt* gene as an example. shRNA is connected by a justice chain, an antisense chain, and a loop that are built in a Valium 10/20 vector. (**B**) Gal4 drives UAS-shRNA production and subsequent activation of the RNAi pathway. (**C**) Real-time quantitative PCR of RNA isolated from *Drosophila* head. The expression of *mtt* RNAi and *mGluR* RNAi transgenes driven by *Actin5c*-Gal4 significantly reduced the mRNA levels of the corresponding genes in adults. Bars represent mean ± standard error of the mean (S.E.M.) for duplicate samples (two independent samples of 30 adult heads). The significance of the difference was analyzed using the one-way analysis of variance (ANOVA) or *t*-test (*** *p* < 0.001; ns, not significant).

In order to obtain a fly model for CSNB, we employed the GAL4/UAS expression system and constructed the UAS-*mtt*/*mGluR* RNAi silencing constructs (derived from the TsingHua Fly Center) (Table 1). The UAS-*mtt*/*mGluR* RNAi transgenes used in subsequent experiments were built by cloning hairpin fragments targeted to *mtt* or *mGluR* into the VALIUM10/20 vector [23]. These constructs rely on GAL4 to induce tissue-specific expression, generating hairpin structures that form siRNA and silencing the expression of *mtt* or *mGluR* in the target tissues (Figure 2A,B). Real-time quantitative PCR demonstrated that GAL4-induced RNAi led to a reduction in the mRNA levels of *mtt* or *mGluR* in the adult fly head with a pan cell driver (*Actin5c*-Gal4) (Figure 2C). Noteworthily, ubiquitous expression of *mtt*-RNAi-1 resulted in nonviable offspring, indicating strong interference by *mtt*-RNAi-1. In contrast, the interference effect of *mtt*-RNAi-1 was not statistically significant (*p* = 0.1746) (Figure 2C).

### 2.3. RNA Interference Targeting mtt Results in Significant Degeneration of Eyes

**Figure 3:**
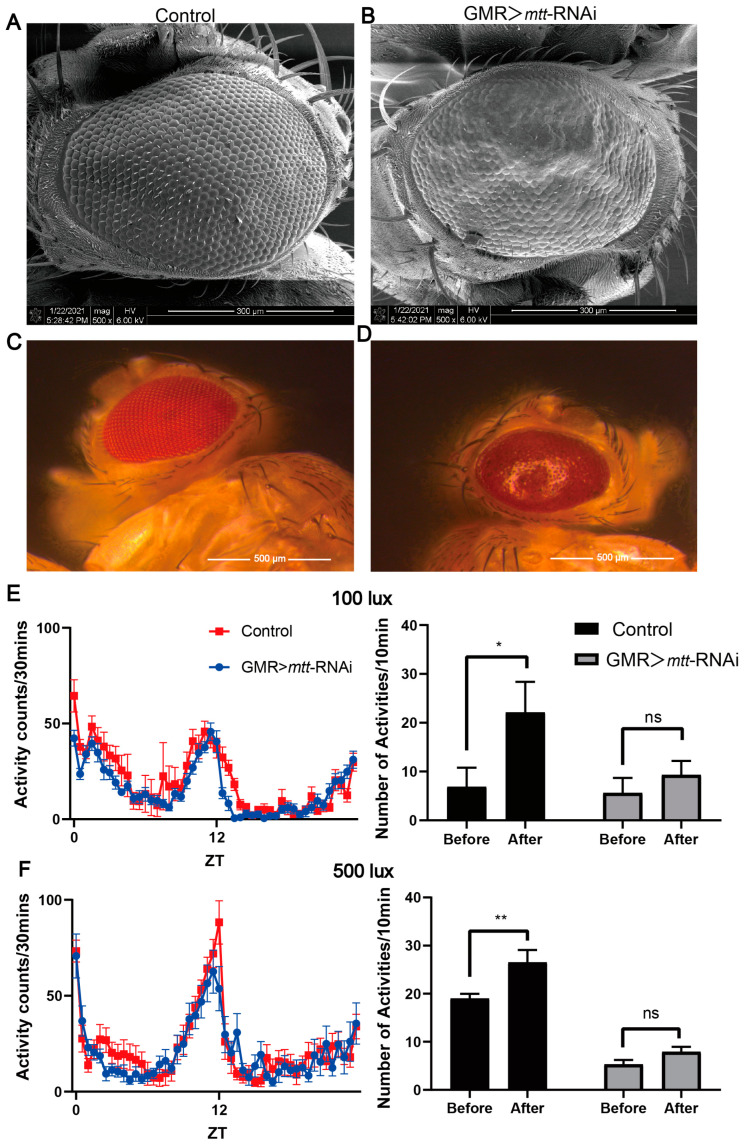
Reduced expression of *mtt* interferes with the visual system of flies. (**A**,**B**) Scanning electron micrographs of 3-to-5-day-old adult eyes showing varying degrees of degeneration. (**A**) Control flies show no eye defects. (**B**) Significantly disrupted eyes in GMR-Gal4 > *mtt*-RNAi flies. (**C**,**D**) Images captured under a stereomicroscope depicting the eyes of *Drosophila* with various genotypes. (**E**) Comparison activity patterns of GMR > *mtt*-RNAi and its control under the light intensity of 100 lux (left panel). Changes in *Drosophila* activity before and after light stimulation under the light intensity of 100 lux (right panel). (**F**) Comparison activity patterns of GMR > *mtt*-RNAi and its control under the light intensity of 500 lux (left panel). Changes in *Drosophila* activity before and after light stimulation under the light intensity of 500 lux (right panel). Bars represent mean ± standard error of the mean (S.E.M.) for duplicate samples (two independent samples of 30 adults). The significance of the difference was analyzed using the 2-way analysis of variance (ANOVA) (* *p* < 0.05; ** *p* < 0.01; ns, not significant). ZT: zeitgeber time.

The main clinical features of CSNB patients and model mice include retinal functional defects. To assess the expression and silencing effects of *mtt*/*mGluR*-RNAi in the eyes, we utilized the eye-specific GAL4 driver, GMR-GAL4 (Glass Multimer Reporter) [25], to induce RNA interference targeting the target genes throughout the entire eye development process. As a control, we chose flies with GMR-GAL4 driving GFP-RNAi, and these control flies exhibited no eye defects (Figure 3A,C). In contrast, the strains with eye-specific expression of *mtt*-RNAi-1 showed disrupted morphological structures of the eyes (Figure 3B,D), characterized by eye malformations such as fused small eyes and the absence of bristles between small eyes. Remarkably, the eye structure was dramatically disrupted in the GMR-GAL4 > *mtt*-RNAi-1 strain, but not in the *mGluR* RNAi strains (Figure S2). To validate whether the visual function of this fly model was affected, we tested their sensitivity to light. Flies were introduced into a locomotor monitor, subjected to overnight dark adaptation, exposed to light stimulation for 10 s one hour prior to light on, and subsequently analyzed for activity during the 10 min before and after light stimulation. At two different light intensities (100 lux and 500 lux), we did not observe any obvious alteration in the activity patterns of flies with GMR-GAL4 > *mtt*-RNAi-1 compared to the control (Figure 3E,F). Interestingly, we found that control flies’ activities were immediately stimulated by light with 100 lux or 500 lux, but the GMR-GAL4 > *mtt*-RNAi-1 flies showed no reaction at all (Figure 3E,F). This indicates that *mtt* play an essential role in both the structure and function of the fly eyes.

### 2.4. Interference with mtt Disrupts the Development of the Fly Eye Disc

**Figure 4:**
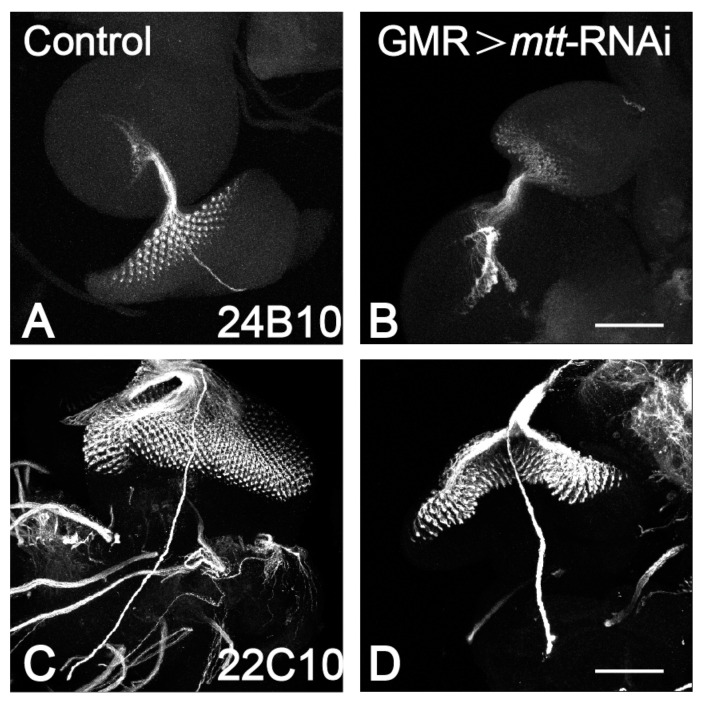
Reduced expression of *mtt* affects the morphology and axonal targeting of the eye disc. Chaoptin (MAb24B10), a marker for the axonal targeting from retina to the optic lobes of the brain. (**A**) In control eye imaginal discs, the retinal axons marked by MAb24B10 innervate the lamina and medulla of the brain. (**B**) GMR > *mtt*-RNAi exhibit severe morphological disruption and axonal abnormalities. Expression of 22C10 in (**C**) Control, (**D**) GMR > *mtt*-RNAi. Scale bar = 100 µm.

Previous studies on mutant flies with loss of *mtt* function only reported the inhibition of their avoidance response to L-Canavanine (a toxic substance) [22]. In this study, we have discovered that silencing *mtt* in eyes also affects the morphology and function of the compound eyes in adult flies. To further understand the potential association between *mtt* and the development of fly eyes, we used 24B10 (Chaoptin, marking photoreceptors and their axons) to label third-instar larvae. In the normal fly eye discs [26], the axons of R1–R6 project to the lamina, while R7–R8 project to the medulla. In comparison to the control group, the eye discs of the GMR > *mtt*-RNAi-1 group exhibit severe morphological disruption and axonal abnormalities (Figure 4A,B). We also used 22C10 for labeling (monoclonal antibodies against the *Drosophila* nervous system) to label larvae eyes [27]. Compared to the control group, the silencing of the *mtt* gene in the eyes shows morphological defects in the eye discs, while the axonal processes are unaffected (Figure 4C,D).

### 2.5. mtt Is Expressed in the Lamina Neurons Rather than Photoreceptor Cells in Adult Flies

**Figure 5:**
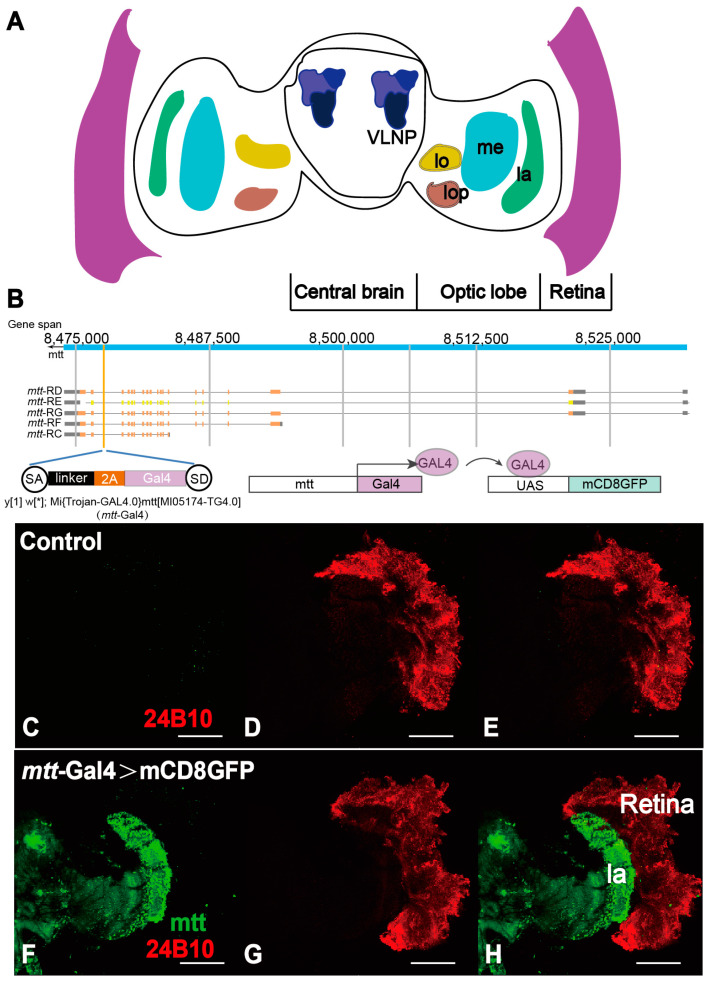
*mtt* is involved in the transmission of the fly visual system and is located in the lamina (la). (**A**) Cartoon graph of the fly brain indicating the different parts of the visual system: eye, lamina (la), medulla (me), lobula (lo), lobula plate (lop), and ventrolateral neuropils (VLNPs). (**B**) Schematic diagram of the principle design of *mtt*-Gal4 and schematic diagram of the subsequent driving mCD8GFP expression. y[1] indicates a yellow mutant allele and w[*] indicates a white mutant allele. (**C**–**E**) Control expressions of (**C**) GFP, (**D**) 24B10, (**E**) Merge. (**F**–**H**) *mtt*-Gal4 > mCD8GFP flies show mtt is located in the lamina (la). Scale bar = 100 µm.

The visual system of the fly consists of the compound eyes, lamina (la), medulla (me), lobula (lo), lobula plate (lop), and ventral lateral neuron processes (VLNP) (Figure 5A). To investigate whether the morphological effects of *mtt* silencing on the compound eyes are directly related to its expression in the retina, we utilized *mtt*-Gal4 to drive the specific expression of green fluorescent protein (GFP) in cells expressing *mtt* (Figure 5B). Additionally, we used the previously mentioned 24B10 antibody as a reference. When dissecting the head, including the compound eye section, we observed that the control group’s 24B10 (photoreceptor cells) was located in the compound eye section, while mtt was widely expressed in the lamina neurons of the fly visual system. The lamina is primarily composed of interneurons, projecting within the optic lobe without leaving the visual ganglia. The cell bodies are located in the lamina cortical region, and the neurons contact individual cartridges, projecting onto the medulla in the retina [27,28,29]. This aligns with the function of the homologous gene GRM6 in mammalian bipolar cells, indicating that mtt plays a transitional role in the visual signal projection in flies.

## 3. Discussion

Currently, research on the pathogenic genes LRIT3, NYX, GRM6, TRPM1, and GPR179 mutations in CSNB is relatively mature. TRPM1 is synthesized in the endoplasmic reticulum and, after modification by the Golgi apparatus, enters the cytoplasm. Studies by Pearring et al. suggest that the proper localization of TRPM1 on ON-BCs dendrites requires the expression of mGluR6/GRM6 and NXY [30]. NXY is located extracellularly, and its interaction with the intracellular PDZ-binding motif of LRIT3 facilitates the proper localization of TRPM1 on the dendrites of photoreceptors and ON-BCs, maintaining a certain level [31]. NXY also plays a role in the synthesis of TRPM1 and its stable expression on the dendrites of ON-BCs [32]. GPR179 is capable of properly positioning the regulator of G protein signaling proteins (RGS), thus activating G proteins. Ray et al. hypothesized that NXY, mGluR6, GPR179, and TRPM1 form a postsynaptic signaling complex, and the LRIT3 protein plays a decisive role in the expression of complex-associated proteins (mGluR6, TRPM1, GPR179, NXY, RGS7, RGS11, R9AP, Gα0, Gβ13, Gβ) [33,34]. Nevertheless, the presence of homologs for these genes in *Drosophila* remains unexplored. Subsequent research endeavors can delve deeper into this matter, seeking to determine whether these genes fulfill specific functions within the *Drosophila* retina.

Gene therapy based on adeno-associated virus (AAV) typically focuses on the relatively accessible photoreceptors and retinal pigment epithelium within the outer retina [35]. However, targeting the elusive bipolar cells in the mid-retina, where defects occur, for the treatment of CSNB remains challenging. Currently, there is no effective treatment for CSNB in humans, but there have been some breakthroughs using gene therapy approaches in animal models. Hasan et al. demonstrated the expression of LRIT3 in rod photoreceptors, and by synaptic interaction, localized the TRPM1 channel to the postsynaptic membrane. Knocking out LRIT3 expression in mice could restore retinal function [36]. Miyadera et al. successfully expressed LRIT3 in bipolar cells of a CSNB model dog by modifying AAV capsid proteins and promoters, leading to significant improvement in visual function [35]. While attempts have been made at gene therapy in CSNB mice with GRM6 mutations, it has not restored normal visual function [37]. Considering the limitations of gene therapy in visual recovery of CSNB mice with GRM6 mutations, it is essential to explore alternative approaches to unmask the pathogenesis of this disease, and we hope to develop more effective treatment methods. In this study, we successfully established a fly model of CSNB, providing a more efficient and economical system for studying the pathogenesis and other factors of this disease. The observed degeneration In the eyes following *mtt* gene knockdown and whether it intensifies with age require further investigation. The flies used for phenotype observation in this study were 3–5 days old, young flies that already exhibited distinct phenotypes. The reported eye disease phenotype related to GRM6-associated CSNB does not intensify with increasing age. Therefore, we believe that this phenotype is less likely to be exacerbated with age.

It is known that mtt, mGluR, and GRM6 are all G protein-coupled receptors (GPCRs), and their amino acids belong to the C family of G protein-coupled receptors (GPCRs). All members of this family exhibit a common structural system characterized by a long N-terminal extracellular domain containing the double-leaf LBP, seven transmembrane domains, and an intracellular C-terminal. This family includes the metabotropic glutamate receptors (mGluRs). In mammals and insects, mGluRs activated by the neurotransmitter glutamate play different roles in the central nervous system. So far, direct homologs of DmXR have only been found in insects. DmXR differs from mGluR in the distal part of the LBP, making it an orphan receptor that is not activated by glutamate. However, research indicates that DmXR and mGluR LBP share critical residues required for binding ligands with amino acid structural characteristics [21]. The *mtt* gene is involved in feeding behavior in adult flies and belongs to the phospholipase C-activated G protein-coupled receptor signaling pathway, located in the dendrites [21,38]. On the other hand, mGluR, serving as a metabotropic glutamate receptor in flies, participates in various processes, including the G protein-coupled glutamate receptor signaling pathway, learning or memory, and synaptic organization [39]. Glutamate and mGluR have been identified as integral elements within the clock circuitry [40] and mushroom bodies [41]. Our study only identified the expression sites of mtt in the visual system of flies. In the future, it will be necessary to also determine the specific expression sites of mGluR to explain why its interference does not affect the normal functioning of the fly eyes.

Our *mtt*-RNAi adult flies exhibited severe disruption in eye morphology, affecting their visual function. Additionally, we have observed significant expression of *mtt* in the lamina neurons of the visual system in the fly brain. This validates that *mtt* is not only involved in adult feeding behavior and response to pesticides but also participates in the phospholipase C-activated G protein-coupled receptor signaling pathway in the visual system of adult flies. When flies are exposed to light, 11-cis-retinal on the retina covalently binds to a lysine in the seventh transmembrane helix of the “rhodopsin” protein (black cylinder). Rhodopsin is approximately 340–500 amino acids long [42]. Upon photon absorption, a twist at the C-11 position extends to produce the all-trans isomer, and the sixth helix of rhodopsin moves to reveal the binding site of the Gα subunit of the trimeric G protein. In flies, the Gα signal is then transmitted to phospholipase C (PLC), which converts phosphatidylinositol 4,5-bisphosphate (PIP2) into inositol 1,4,5-trisphosphate (IP3) and diacylglycerol (DAG) [43]. Confirming the role of mtt in mediating the response of this signaling pathway requires additional exploration in future studies. The inclusion of ERG detection, indeed, would enhance the robustness of the data presented in this article.

In summary, we have successfully generated a fly model of CSNB through RNAi-mediated silencing of *mtt* with eye-specific expression. Similarly to CSNB patients with corresponding GRM6 mutations and mouse models, our fly model exhibited impaired visual function. The interference with *mtt* expression disrupts the phospholipase C signaling pathway in the visual system, leading to abnormal eye development. However, the survival and other phenotypes of our model have not been assessed yet.

## 4. Materials and Methods

### 4.1. Fly Stocks

The flies were kept at 25 °C unless otherwise stated. The following lines were obtained from the Bloomington *Drosophila* Stock Center: UAS-mCD8::GFP (#5137), UAS-GFP-RNAi (#9331), and *mtt*-Gal4 (#66799). The following lines were obtained from the TsingHua Fly Center: UAS-*mGluR*-RNAi-1 (#THU2115), UAS-*mGluR*-RNAi-2 (#THU5288), UAS-*mtt*-RNAi-1 (#THU5594), UAS-*mtt*-RNAi-2 (#THU0827).

### 4.2. Quantitative Real-Time PCR

Total RNA was isolated from the head of adult flies using TRIzon reagent (CW bio, Beijing, China, Catalog No. CW0580). For each extraction, a total of 30 adult heads were dissected in 500 μL of TRIzon reagent. Duplicate or triplicate samples were used for each genotype. After RNA isolation, cDNA was synthesized from 1 μg of total RNA using the cDNA prepared using HiScript III RT SuperMix (Vazyme, Nanjing, China, Catalog No. R323). qPCR was performed using Eastep qPCR Master Mix Kit (Promega, Beijing, China, Catalog No. LS2062) on a LightCycler 96 (Roche, Rotkreuz, Switzerland). All protocols were performed according to the manufacturer’s instructions. The following primers were used (*actin5C* was used as an internal control): *mtt*-f: 5′-GCAATCCCTGGTTTGTGGAAT-3′, *mtt*-r: 5′-GAAAGTCGCTCCTTTGTGGTG-3′, *mGluR*-f: 5′-AACGGAACAATTTTAGTCGTCGT-3′, *mGluR*-f: 5′-GCAGAGAAACAGACACTGAATCC-3′, *actin5C*-f:5′-CAGAGCAAGCGTGGTATCCT-3′, *actin5C*-r: 5′-CTCATTGTAGAAGGTGTGGTGC-3′.

### 4.3. Acquisition of Protein Three-Dimensional Structure

The protein three-dimensional structures were obtained from the AlphaFold Protein Structure Database [44,45]. The three structure files acquired were as follows: hGRM6 (AF-O15303-F1-model_v4), mGluR (AF-P91685-F1-model_v4), and mtt (AF-Q70GQ8-F1-model_v4). Molecular graphics and analyses were conducted using UCSF Chimera 1.17.1, a software developed by the Resource for Biocomputing, Visualization, and Informatics at the University of California, San Francisco [46].

### 4.4. Scanning Electron Microscopy

To more clearly detect morphological changes in fly eye cells after eye-specific interference with the mtt gene, we conducted scanning electron microscopy. Briefly, 2–3-day-old flies were killed in a −80 °C fridge, then air-dried at room temperature for a few days, and subsequently coated in gold for photography on a field emission scanning electron microscope (FEI, Helios G4 CX, ThermoFisher Scientific, Waltham, MA, USA) at approximately 110X magnification.

### 4.5. Photostimulus Response

In order to assess whether flies exhibited reduced sensitivity to light stimulation after eye-specific interference with the mtt gene, three-to-five-day-old male flies were used for locomotor behavior analysis on *Drosophila* Activity Monitor 2 (DAM2, Trikinetics, Waltham, MA, USA). Adult flies were fed 5% sucrose and 1% agar. Light conditions were set at 12 h light and 12 h dark (12L:12D), and the incubator temperature was maintained at 25 °C. After 2 days, one hour before turning on the light the next day, light stimulation was performed for 10 s to observe their activity. Activity data for 10 min before and after light stimulation were used for analysis. The light source used was a full-spectrum LED white light, with two sets of light intensity conditions, namely 100 lux and 500 lux, being configured.

### 4.6. Immunohistochemistry

Eye-antennal discs from wandering third instar larvae were dissected, and fixed in 4% paraformaldehyde in Phosphate-Buffered Saline (PBS), and stained following the protocol. The primary antibodies used were mouse anti-Chaoptin (MAb24B10) (1:100, DSHB #24B10, Iowa, IA, USA), mouse anti-22C10 (1:100, DSHB #22C10, Iowa, IA, USA) and rabbit anti-GFP (1:1000, ThermoFisher Scientific #A11122, Waltham, MA, USA), while the secondary antibody were Alexa Fluor 568 goat anti-mouse antibody (1:1000, ThermoFisher Scientific #A11031, Waltham, MA, USA) or Alexa Fluor 488 goat anti-rabbit antibody (1:1000, ThermoFisher Scientific # A11008, Waltham, MA, USA).

The brains of three-to-five-day-old mtt-Gal4 > UAS-mCD8::GFP flies were dissected with their retina preserved. At first, the whole flies were fixed in 4% formaldehyde at 25 °C for 2 h. Then, the dissected brains were washed in PBT (PBS containing 0.2% Triton X-100) and blocked in 5% goat serum in PBT (PBST) for 30 min at room temperature. The primary antibodies were used to incubate the samples overnight at 4 °C. Finally, samples were washed three times with PBT and incubated overnight with Alexa Fluor 488-labeled goat anti-rabbit or Alexa Fluor 568-labeled goat anti-mouse secondary antibody overnight at 4 °C. Images of mounted samples were captured under confocal microscope (Leica TCS SP5, Wetzlar, Germany).

### 4.7. Statistical Analysis

GraphPad Prism 7 was used for all statistical analyses. To compare 2 groups, Student t test was used, and to compare multiple groups, an ordinary one-way analysis of variance (ANOVA) was used. For ordinary one-way ANOVA, Tukey’s multiple comparison tests were used.

## Figures and Tables

**Table 1:** Overview of the RNAi-silenced fly strains used in the study.

Num	TRiP#	Gene Symbol	Vector	Hairpin ID	Hairpin Location	Reference
1	JF01958	*mGluR*	VALIUM10	TR01912P.1	attP2	[24]
2	HMS02201	*mGluR*	VALIUM20	SH03131.N	attP40	
3	HMS02793	*mtt*	VALIUM20	SH06282.N	attP40	
4	HMS00367	*mtt*	VALIUM20	SH00643.N	attP2	

## Data Availability

The data presented in this study are available on request from the corresponding author.

## Supplementary Materials

The following supporting information can be downloaded at: https://www.mdpi.com/article/10.3390/ijms25020899/s1.
