# Supplementary material for: A Drosophila Model Reveals the Potential Role for mtt in Retinal Disease
Source: Int J Mol Sci. 2024 Jan 11;25(2):899. doi: 10.3390/ijms25020899 (PMC10815649; doi:10.3390/ijms25020899)
Supplement: Supplementary file 1 [file ijms-25-00899-s001.zip › ijms-2795064-supplementary.pdf]

```
mtt         mttanivaapaaptispaitstatriggkavptprllgsgaanva-----qilaksldsp 55
mGluR       -------------------------------mkqknn-ngtilvvvmvlswsrvvdlksp 28
Human       ------------------------------marprra-repllvallplawlaqagla-- 27
                                              :        *.          .*

mtt         spssssgwpvkhaavlegdvilgglmmvhsred-sitcgpimpqggiqaleamlytldqv 114
mGluR       snt---htqdsvsvslpgdiilgglfpvhekge-gapcgpkvynrgvqrleamlyaidrv 84
Human       --------raagsvrlaggltlgglfpvhargaagracgqlkkeqgvhrleamlyaldrv 79
                        :. * *.: ****: ** :   .   **   : *:: ******::*:*

mtt         nk-qqllpnvtlgahllddcdkdtyglemavdfikgsisn---iddaeyhcnktq----- 165
mGluR       nndpnilpgitigvhildtcsrdtyalnqslqfvraslnn---ldtsgyecadgsspqlr 141
Human       nadpellpgvrlgarlldtcsrdtyaleqalsfvqalirgrgdgdevgvrcpggvppl-r 138
            *   ::**.: :*.::** *.:***.*: ::.*::. : .    *    .*

mtt         ---vrkvisgvvgaassvtsiqvanllrlfripqvsyfstspelsnkqrfeyfsrtipsd 222
mGluR       knassgpvfgviggsyssvslqvanllrlfhipqvspastaktlsdktrfdlfartvppd 201
Human       -pappervvavvgasassvsimvanvlrlfaipqisyastapelsdstrydffsrvvppd 197
                   : .*:*.: * .*: ***:**** ***:*  **:  **:. *:: *:*.:* *

mtt         hyqvkamveivkrmgwsyvsiiyeesnygikafeeleell-arhniciaikeklvkdsgv 281
mGluR       tfqsvalvdilknfnwsyvstihsegsygeygiealhkea-ternvciavaekvps---a 257
Human       syqaqamvdivralgwnyvstlasegnygesgveafvqisreaggvciaqsikipr---e 254
             :*  *:*:*:: :.*.*** : .*..**  ..* : :      .:***   *:

mtt         aediaydnivqklltkprargaiifgsdqevrqvmravrranatgsfswigsdgwsarnl 341
mGluR       addkvfdsiisklqkkpnargvvlftraedarrilqaakranlsqpfhwiasdgwgkqqk 317
Human       pkpgefskvirrlmetpnargiiifaneddirrvleaarqanltghflwvgsdswgakts 314
             .   :..:: :*  .*.*** ::*   :: *:::.*.::** :  * *:.**.*. :

mtt         vsddyepevegtlsvqpqanpvrgfeeyflsltvennqrnpwfvefwedhfqcrypgsts 401
mGluR       llegledjaegaitvelqseiiadfdrymmqltpetnqrnpwfaeywedtfncvltslsv 377
Human       pilsledvavgaitilpkrasidgfdqyfmtrslennrrniwfaefweenfncklts sgt 374
               . *  . *::::  :   : .*:.*::  : *.*:** **.*:**: *:*    .

mtt         tpynn-------------ytkqcttkerlsrqn-tdfedqlqfvsdavmafayalrdmhr 447
mGluR       kpdtsnsanstdnkigvkaktecddsyrlsekvgyeqesktqfvvdavyafayalhnlhn 437
Human       qsdds--------------trkctgeerigrdstyeqegkvqfvidavyaiahalhsmhq 420
                .              . :*  . *:...   : *.: *** *** *:*:**:.:*.

mtt         dlcgggp--------------------------slceamkptkgad-llkylrkvefegl 480
mGluR       drcntqsdqttetrkhlqsesvwyrkistdtksqacpdmanydgkefynnyllnvsfidl 497
Human       alcpght--------------------------glcpameptdgrm-llqyiravrfngs 453
              *                                *  *   .*     :*:  * * .

mtt         sgdefrfdgngdgparyniihfkqsqa--gqyhwvkvgeytegelrlnmtevkfkrlspk 538
mGluR       agsevkfdrqgdglarydilnyqrqens-sgyqykvigkwfng-lqlnsetvvwnket-e 554
Human       agtpvmfnengdapgrydifqyqatngsassggyqavgqwaet-lrldvealqwsgdphe 512
            :*  . *: :**. .**:*::::  :   .   :  :*:: :  *:*:   : :.    :

mtt         ppesvcslpclvgqakkyvegesccwhcfncttyqirhpddethcklcklgtlpdahkqy 598
mGluR       qptsacslpcevgmikk-qqgdtccwicdscesfey--vydeftckdcgpglwpyadkls 611
Human       vpsslcslpcgpgerkkmvkgvpccwhceacdgyrf--qvdeftceacpgdmrptpnhtg 570
             * * *****  *  **  :*  *** *  *  :.     **  *: *  .  *  .:

mtt         crpipeiylrpesawaigamafsatgilvtlfvmgvfvrhndtpivrasgrelsyillag 658
mGluR       cyaldiqymkwnslfalipmaiaifgialtsivivlfaknhdtplvrasgrelsytllfg 671
Human       crptpvvrlswsspwaappllllavlgivatttvvatfvrynntpivrasgrelsyvlltg 630
            *       :  .* :*   : ::  **  *  *:  *.: ::**:********** ** *

mtt         ifmcygvtfalvlkptnivcaiqrfgvgfcftvvyaalltktnriarifkagkqsakrps 718
mGluR       ilvcycntfaliakptigscvlqrfgigvgfsiiysalltktnrisrifhsasksaqrlk 731
Human       ifliyaitflmvaepgaavcaarrlflglgttls salltktnriyrifeqgkrsvtppp 690
            *:: *  ** ::  *    *. :*: :*.  :: *:********* ***. ..:*.

mtt         fispksqlvicaclvsvqilingvwmviapshamhhypt-------rednllvcdsyida 771
mGluR       yispqsqvvittsliaiqvlitmiwmvveppgtrfyypd-------rrevilkck-iqdm 783
Human       fisptsqlvitfsltslqvvgmiawlgarpphsvidyeeqrtvdpeqargvlkcd-msdl 749
            :*** **:**  .* ::*::    *:   *  :   *         :   :* *.   *

mtt         symiafsypiflivictvyavltrkipeafneskhigftmyttcviwlafvplyfgtanh 831
mGluR       sflfsqlynmiliticityaiktrkipenfneskfigftmyttciiwlafvpiyfgtgns 843
Human       sligclgysllmvtctvyaikargvpetfneakpigftmyttciiwlafvpiffgtaqs 809
            * : .  * ::*:. **:**: :* :** ***:* *********:*******::***.:

mtt         vp---lritsmsvtislsasvtiaclfspklyiilirpernvrqsmmpprygnmh----- 883
mGluR       ye---vqtttlcisislsasvalvclyspkvyilvfhpdknvrkltmnstvyrrsaaava 900
Human       aekiyiqtttltvslslsasvslgmlyvpktyvilfhpeqnvqkrkrslkatstv----- 864
                 :: *:: :::******::  *: ** *:::::*::**::

mtt         ----------rtagtgpssmmaaavvtaatcaqeekiqkhitptnten------------ 921
mGluR       qgaptssgysrthapgtsaltggavgtnass---stlptqnsphldeasaqtnvahktng 957
Human       -aappkgedaeahk---------------------------------------------- 877
                      .:

mtt         ------slktkklcematqtissiitsldinaynqipyadqyvpnnattatttptdkgsg 975
mGluR       eflpevgervepichivnk----------------------------------------- 976
Human       ------------------------------------------------------------ 877

mtt         ngnsnsnsntngssnsnsnsngdgdvateqvvannnkinqrqhgqpavafaitstnnhis 1035
mGluR       ------------------------------------------------------------ 976
Human       ------------------------------------------------------------ 877

mtt         gpaatitttaatgettteal vatittplatvdgsetmsvapnnghgnngsqrppvlqtnl 1095
mGluR       ------------------------------------------------------------ 976
Human       ------------------------------------------------------------ 877
```

Figure S1. Alignment of human GRM6 and fly mtt/mGluR proteins. '*' indicates positions, which have a single, fully conserved residue; ':' indicates that one of the following 'strong' groups is fully conserved: STA, NEQK, NHQK, NDEQ, QHRK, MILV, MILF, HY, FYW; '.' indicates that one of the following 'weaker' groups is fully conserved: CSA, ATV, SAG, STNK, STPA, SGND, SNDEQK, NDEQHK, NEQHRK, FVLIM, and HFY.

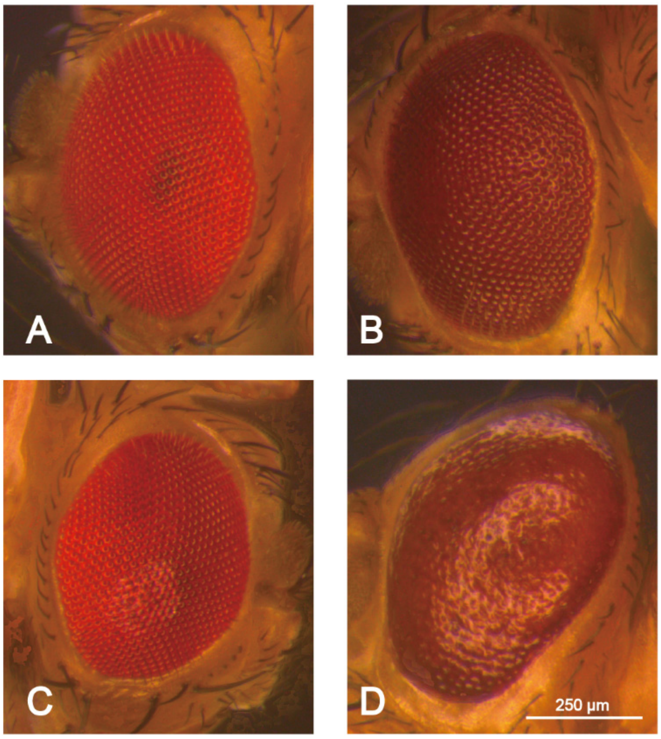

Figure S2. Images captured under a stereomicroscope depicting the eyes of *Drosophila* with various genotypes. (A) GMR-Gal4>*mGluR*-RNAi-1 (#THU2115). (B) GMR-Gal4>*mGluR*-RNAi-2 (#THU5288). (C) GMR>*mtt*-RNAi-2 (#THU0827). (D) GMR>*mtt*-RNAi-1 (#THU5594).
